# A 5 *g* Inertial Micro-Switch with Enhanced Threshold Accuracy Using Squeeze-Film Damping

**DOI:** 10.3390/mi9110539

**Published:** 2018-10-23

**Authors:** Yingchun Peng, Guoguo Wu, Chunpeng Pan, Cheng Lv, Tianhong Luo

**Affiliations:** College of Mechanical and Electrical Engineering, Chongqing University of Arts and Sciences, Chongqing 402160, China; 20140014@cqwu.edu.cn (G.W.); 20170118@cqwu.edu.cn (C.P.); 20170017@cqwu.edu.cn (C.L.)

**Keywords:** MEMS (micro-electro-mechanical system), inertial switch, acceleration switch, threshold accuracy, squeeze-film damping

## Abstract

Our previous report based on a 10 *g* (gravity) silicon-based inertial micro-switch showed that the contact effect between the two electrodes can be improved by squeeze-film damping. As an extended study toward its potential applications, the switch with a large proof mass suspended by four flexible serpentine springs was redesigned to achieve 5 *g* threshold value and enhanced threshold accuracy. The impact of the squeeze-film damping on the threshold value was theoretically studied. The theoretical results show that the threshold variation from the designed value due to fabrication errors can be reduced by optimizing the device thickness (the thickness of the proof mass and springs) and then establishing a tradeoff between the damping and elastic forces, thus improving the threshold accuracy. The design strategy was verified by FEM (finite-element-method) simulation and an experimental test. The simulation results show that the maximum threshold deviation was only 0.15 *g*, when the device thickness variation range was 16–24 μm, which is an adequately wide latitude for the current bulk silicon micromachining technology. The measured threshold values were 4.9–5.8 *g* and the device thicknesses were 18.2–22.5 μm, agreeing well with the simulation results. The measured contact time was 50 μs which is also in good agreement with our previous work.

## 1. Introduction

Inertial micro-switches based on MEMS (micro-electro-mechanical system) technology have been widely used for acceleration sensing applications [1,2] due to their small size, high integration level, and low or even no power consumption [3,4]. The inertial micro-switches are typically designed with a proof mass that is anchored to a substrate through flexible springs. The proof mass serves as a moveable electrode, and it is separated by a certain distance from a fixed electrode on the substrate. At a pre-selected threshold acceleration, the moveable electrode moves toward the substrate and it comes into contact with the fixed electrode, turning on the switch and triggering the external circuit. Thus, the inertial micro-switches require a reliable contact effect of the two electrodes, such that the turn-on signal can be recognized by the external circuit. From the perspective of application convenience, since most of the switches are mass produced in the industry sector, a high degree of device-to-device threshold uniformity of the same production batch is needed. As such, a high threshold accuracy is also essential for the inertial micro-switches.

Since the first inertial micro-switch was reported in 1972 [5], a great number of inertial micro-switches based on various working mechanisms and manufacturing methods have been developed. However, most of the switches reported in the past have been mainly designed to improve the contact effect and the threshold accuracy is rarely considered. This is because the inherent issues of the methods employed to improve the contact effect usually lead to a low threshold accuracy.

The main methods to improve the contact effect of the inertial micro-switches are designing the switches with a keep-close function, or flexible electrodes. The switches with a keep-close function can keep it closed after the acceleration event is over, thus improving the contact reliability. However, the keep-close function requires special constructions, such as the hook-shaped electrodes of the latching switches [6,7,8], the V-shaped beams of the bi-stable switches [9,10], and the valve-channel of the micro-fluidic switches [11,12], complicating the structure topology or working mechanism or fabrication method, thus reducing the threshold accuracy, as shown in Table 1. The latching switch with a 50.59 *g* designed threshold value in Ref. [7], for instance, was first switched on when the applied acceleration was between 28 *g* and 43.7 *g*, and it was completely closed when a higher acceleration was applied. This is due to the collision and friction contact process of the two hook-shaped electrodes.

The contact effect of the inertial micro-switches with flexible electrodes can be improved by the deformation of the flexible electrodes during the contact process. In this case, the contact time of the two electrodes is generally longer than 50 μs [13,14]. This kind of switch is usually fabricated by a multi-layer nickel-electroplating process, based on the surface micromachining technology, since the conventional bulk silicon micromachining technology mainly results in rigid structures. However, the often-repeated electroplating processes might cause unexpected fabrication errors such as dimension variations, an inhomogeneous Young’s modulus, and structural deformation induced by residual stresses between each electroplating layer, leading to a threshold deviation from the designed value [4,13,14,15,16,17], as shown in Table 1. In Ref. [15], the measured threshold of the switch with 240 *g* designed threshold was 288 *g*, and in Ref. [16], the actual thresholds increased from 32 *g* to 38 *g*, while the intended target was 38 *g*. The multi-layer electroplating process is not applicable to the case of the switches with a designed threshold value below 10 *g* (also named as low-g switch in this paper) because the threshold deviation may be more seriously caused by the fabrication errors. According to the static equilibrium equation $a_{th}=kx_{0}/m$ (where $a_{th}$ is the threshold acceleration, k is the spring constant, $x_{0}$ is the distance between the two electrodes, and m is the mass of the proof mass), a low-g switch requires flexible springs and large proof mass, because the minimum size of $x_{0}$ is usually limited by the fabrication process. The large proof mass should be fabricated by a great number of electroplating processes, resulting in serious fabrication errors.

In recent years, several researchers have paid great attention to improving the threshold accuracy of the inertial micro-switches, as shown in Table 1. McNamara and Gianchandani [18] presented an array redundancy design of the inertial micro-switch to broaden the sensing range of acceleration (10–150 *g* in 10 *g* increments) and allow fault latitude, wherein multi switches at each threshold level were employed. By weighting the measured results on the majority status of these redundant switches, the measured thresholds were 80–90% of the target values. Jr and Epp [19] proposed a stochastic dynamics model to modify the device dimensions based on the experimental results, reducing the threshold deviation caused by the fabrication errors. Currano et al. [4] demonstrated an inertial micro-switch that could detect identical accelerations in the *x*, *y*, and *z* axes using a single mass/spring assembly. To reduce the threshold deviation due to the fabrication errors, they modified the 2 μm width spring (the original designed value) to 5 μm, and changed the spring lengths to tune the in-plane (*x*/*y*) thresholds to the target acceleration levels. Then, the in-plane threshold values were generally close to the designed values, but the thresholds in the *z*-axis were much lower than the target levels (~10–40 *g*, as opposed to ~90–230 *g*). Du et al. [20] modified the device thickness of the switch, based on the sizes of the pre-fabricated structure components. The designed and measured thresholds were 38 *g* and 35–40 *g*, respectively. Zhang et al. [21] fabricated a 5.5 *g* inertial micro-switch on a SOI (silicon-on-insulator) wafer to accurately define the device thickness, thus improving the threshold accuracy. The measured threshold values were 4.77–5.97 *g*.

In our previous report [22], an inertial micro-switch with a threshold value of 10 *g* and a high damping ratio of 2 was presented based on a typical silicon-on-glass process. The contact effect (40 µs contact time) was significantly improved using the squeeze-film damping effect compared with the typical switches with rigid electrodes (the contact time usually less than 20 µs). In this paper, the impact of the squeeze-film damping on the threshold acceleration was studied by theoretical analysis, FEM (finite-element-method) simulation and experimental test. The study was implemented based on our previous device structure but with a lower threshold value of 5 *g*. The experimental results show that squeeze-film damping can not only prolong the contact time, but also improve the threshold accuracy. The study is significant for the applications of the inertial micro-switches, where low-g-sensing, long contact time, and high threshold accuracy are required.

## 2. Theory and FEM Simulation

### 2.1. Device Structure

**Figure 1:**
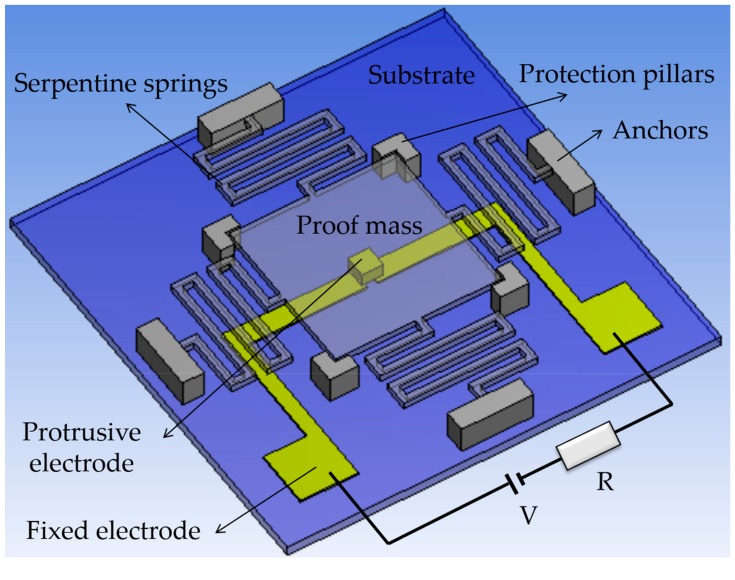
Scheme of the designed inertial micro-switch.

The switch consists of a proof mass that is suspended by four flexible serpentine springs, which serves as the sensing element moving toward the substrate to sense out-of-plane acceleration. The vertical direction sensitivity enables the switch to employ the squeeze-film damping effect, since the slid-film damping effect involved by laterally driven switches is so weak that it is usually neglected. A small size of protrusion positioned at the bottom center of the proof mass is defined as the movable contact electrode, reducing the contact area. Two separated metal strips on the substrate serve as the double-contact-configuration fixed electrode. When an environmental acceleration exceeding the preset threshold is applied to the switch in the sensitive direction, the proof mass moves toward the substrate, traveling the electrode gap and making the movable electrode contact with the fixed electrode, thus turning the switch on. The movement of the proof mass in the horizontal insensitive directions is limited by four fixed pillars. Figure 1 shows a sketch of the designed switch, wherein the proof mass and springs are set as transparent structures to display the two electrodes under them.

According to the static equilibrium equation mentioned in the introduce section, the electrode gap height ($h_{e}$) was set to 1 μm, which is near our process limit to reduce the required volume of the proof mass for a low threshold value. In this case, the width and length of the proof mass were both set to 2300 μm. The four flexible serpentine springs with a 30 μm width have a much lower equivalent spring constant than the typical cantilever beam. This structure feature enables the switch to easily respond to the target threshold of 5 *g*. The distance between the proof mass and the substrate ($h_{a}=35\;\mu m$) was designed to achieve the required damping ratio (ca. 2.1) by changing the height of the protrusion ($h_{p}$) when the other structure parameters were determined. Due to the small size of the protrusion, the impact of changing $h_{p}$ on the equivalent mass of the proof mass can be ignored.

The thickness of the proof mass is designed to be identical to that of the springs ($t_{b}$) for facilitating fabrication. More importantly, in order to reduce the threshold deviation due to fabrication errors, $t_{b}$ was defined as a crucial dimension, and then theoretically studied to establish a tradeoff between the damping and elastic forces that the switch is subjected to, while operating under an over-damping condition (as explained later).

### 2.2. Theoretical Analysis of the Threshold Accuracy

As a typical inertial sensor, the inertial micro-switch can be modeled by a mass-spring-damping system to represent the mechanical behavior of the device. Considering that the acceleration signal applied to the switch in practical work is a half-sine wave, the governing mechanical equation is:

(1) $$m\ddot{x}+c\dot{x}+kx=ma\sin\omega t,$$

where x is the relative displacement of the proof mass with respect to the substrate, m is the proof mass, c is the squeeze-film damping viscous coefficient, k is the equivalent elastic stiffness of the four serpentine springs, ω is the angular frequency of the acceleration, a is the amplitude of the acceleration, and its minimum value that can close the switch is regarded as the threshold acceleration ($a_{th}$).

The solution to Equation (1) given in [23] consists of two parts: the transient item, which will decrease exponentially with time determined by the damping condition, and the steady-state item, wherein the oscillation of the proof mass is the same frequency as that of the applied acceleration. Considering the over-damping-condition design in this paper, the steady-state item is primary, and the transient item is second of the solution. As such, the solution to Equation (1) given in [23] can be simplified as:

(2) $$x(t)=\frac{a\sin(\omega t-\phi )}{\sqrt{{\left(\omega_{n}{}^{2}-\omega^{2}\right)}^{2}+{\left(c\omega /m\right)}^{2}}},$$

where $\omega_{n}=\sqrt{k/m}$ is the natural angular frequency of the switch, and ϕ is the displacement phase of the proof mass with respect to the substrate. Then, the threshold acceleration can be represented in Equation (3), according to its own definition as mentioned above:

(3) $$a_{th}=\sqrt{{\left(\omega_{n}{}^{2}-\omega^{2}\right)}^{2}+{\left(c\omega /m\right)}^{2}}\cdot h_{e}.$$

In fact, $\omega_{n}$ is usually higher than ω to minimize the threshold discrepancy of the switch, due to the duration of the input acceleration [24]; thus, Equation (3) can be rewritten as:

(4) $${\tilde{a}}_{th}\propto \sqrt{{\left(k/m\right)}^{2}+{\left(c\omega /m\right)}^{2}},$$

where ${\tilde{a}}_{th}$ is the value of $a_{th}$ normalized by $h_{e}$, i.e., ${\tilde{a}}_{th}=a_{th}/h_{e}$. Substituting the expressions of k (which is derived by equivalently taking the four serpentine springs as a cantilever beam) in Equation (5) and c in Equations (6) [25] into (4), the expression of the normalized threshold is rewritten in Equation (7):

(5) $$k=\frac{Ew_{b}t_{b}{}^{3}}{4l_{b}{}^{3}},$$

(6) $$\begin{array}{c} c=\frac{\mu l_{m}w_{m}{}^{3}}{h_{a}{}^{3}}\gamma ,\\ \gamma =\left\{1-\frac{192}{\pi^{5}}\frac{w_{m}}{l_{m}}\sum_{n=1,3,5,}^{\infty }\frac{1}{n^{5}}\tanh\left(\frac{n\pi l_{m}}{2w_{m}}\right)\right\}, \end{array}$$

(7) $${\tilde{a}}_{th}\propto \sqrt{{\left(\frac{Ew_{b}t_{b}{}^{3}}{4l_{b}{}^{3}\rho l_{m}w_{m}t_{m}}\right)}^{2}+{\left(\frac{\mu w_{m}{}^{2}}{h_{a}{}^{3}\rho t_{m}}\gamma \omega \right)}^{2}},$$

where E and ρ are the Young’s modulus and the density of silicon, respectively; $l_{b}$, $w_{b}$, and $t_{b}$ are the length, width, and thickness of the equivalent cantilever beam, respectively; $l_{m}$, $w_{m}$, and $t_{m}$ are the length, width, and thickness of the proof mass, respectively; μ is the viscosity coefficient of air, γ is a correction factor determined by $w_{m}/l_{m}$, and is equal to 0.42 when $w_{m}=l_{m}$.

From the perspective of fabrication, the spring thickness (20 μm in this paper) is much thinner than the silicon wafer (ca. 500 μm thick), and such that it is defined by a several hours of deep back-etch fabrication process (a KOH etching process for low cost and high etch rate). Due to the long duration process, the thickness non-uniformity within the wafer may become obvious induced by several factors, such as the hydrogen generation, and the diffusion of the etchant and reaction products (e.g., the maximal height difference over the back-etched surface of a 18 μm thick sieve is 4.6 μm [26]). Therefore, the variation of the spring thickness due to the fabrication errors is usually larger than that of the spring width in the similar size. Moreover, according to Equation (5), the changing of the spring thickness has greater influence on the spring stiffness than that of the spring width, thus leading to a larger threshold deviation of the switch. Therefore, we define the spring thickness $t_{b}$ as the crucial dimension that may cause the primary threshold deviation to the switch, due to the fabrication errors. For facilitating design and manufacturing, the thickness of the proof mass $t_{m}$ is set to be equal to $t_{b}$, and Equation (7) can then be further rewritten as:

(8) $${\tilde{a}}_{th}\propto \sqrt{\eta_{1}^{2}\cdot t_{b}^{4}+\eta_{2}^{2}/t_{b}^{2}},$$

where $\eta_{1}=Ew_{b}/4l_{b}{}^{3}\rho l_{m}w_{m}$ and $\eta_{2}=\mu w_{m}{}^{2}\gamma \omega /h_{a}{}^{3}\rho$ can be regarded as constants when the structure parameters of the switch have been determined, except for $t_{b}$. Equation (8) indicates that $\eta_{1}^{2}\cdot t_{b}^{4}$ and $\eta_{2}^{2}/t_{b}^{2}$ change in opposite directions with $t_{b}$, meaning that there should be a specific interval value of $t_{b}$ in which the sum of $\eta_{1}^{2}\cdot t_{b}^{4}$ and $\eta_{2}^{2}/t_{b}^{2}$ keeps relatively stable, and then ${\tilde{a}}_{th}$ as well. In this case, the threshold deviation can be significantly reduced, despite the variations of $t_{b}$ caused by fabrication errors.

This design strategy can be explained as follows: (i) according to Equation (1), the forces that the switch is subjected to while operating contain the external force masinωt, the inertial force $m\ddot{x}$, the squeeze-film damping force $c\dot{x}$, and the elastic force kx; (ii) normalizing the four forces by m, then $a_{th}(\omega )$ (its value is influenced by the angular frequency of the acceleration ω), c/m and k/m represent the threshold acceleration, the coefficients of the damping force and elastic force, respectively, and the coefficient of the inertial force is a constant that has no effect on the threshold deviation; (iii) subsequently, the threshold is mainly proportional to the sum of the damping and elastic forces; (iv) according to Equations (5) and (6) and the design of $t_{b}=t_{m}$, k/m and c/m are proportional to $t_{b}{}^{2}$ and $t_{b}{}^{-1}$, respectively. Then, k/m increases and c/m decreases with increasing $t_{b}$, and the two items will be equal for a specific value of $t_{b}$ (labeled as $t_{b}{}^{\prime }$); (v) at $t_{b}<t_{b}{}^{\prime }$, along with the increase of $t_{b}$, the increment of k/m is smaller than the decrement of c/m, thus decreasing the threshold value, and it is the opposite at $t_{b}>t_{b}{}^{\prime }$. (vi) When the value of $t_{b}$ changes around that of $t_{b}{}^{\prime }$, the absolute variations of k/m and c/m are roughly equal; i.e., establishing a tradeoff between the damping and elastic forces, thus keeping the threshold value relatively maintained. It should be noted that this design strategy is only valid when (1) the squeeze-film damping condition is over-dampened such that the damping force is large enough to be comparable with the elastic force, and (2) the value of $t_{b}$ is optimized.

### 2.3. FEM Simulation

FEM transient analysis (ANSYS Workbench, 15.0, ANSYS Inc., Pittsburgh, PA, US) was performed to obtain the proper value of $t_{b}$. The FEM model was meshed by the method of Hex Dominant. The end sections of the four suspended springs were constrained to be zero in all degrees of freedom. In the analysis settings, the damping effect was represented by the so called Rayleigh damping coefficients—the alpha damping coefficient $\alpha =2\zeta \omega_{n1}\omega_{n2}/(\omega_{n1}+\omega_{n2})$ and the beta damping coefficient $\beta =2\zeta /(\omega_{n1}+\omega_{n2})$, where $\zeta =c/2m\omega_{n}$ is the squeeze-film damping ratio, $\omega_{n1}$ and $\omega_{n2}$ are the first and second order natural angular frequencies of the switch, respectively [27]. The frequency of the applied half-sine wave is 500 Hz, i.e., 1 ms duration acceleration, which is lower than that of the designed switch (808 Hz) such that it is enough for explaining the design strategy. The total step number of the acceleration load is 30, and the numbers of the substep (including initial substep, minimum substep, and maximum substep) are 4, 2, and 6, respectively, which are enough for guaranteeing computational accuracy. During the transient analysis, by presetting the electrode-gap height $h_{e}$, the required acceleration amplitude (i.e., the threshold acceleration) applied to the switch for different values of $t_{b}$ can be obtained. The main geometric parameters and the material properties of the switch for the FEM study are shown in Table 2 and Table 3, respectively.

**Figure 2:**
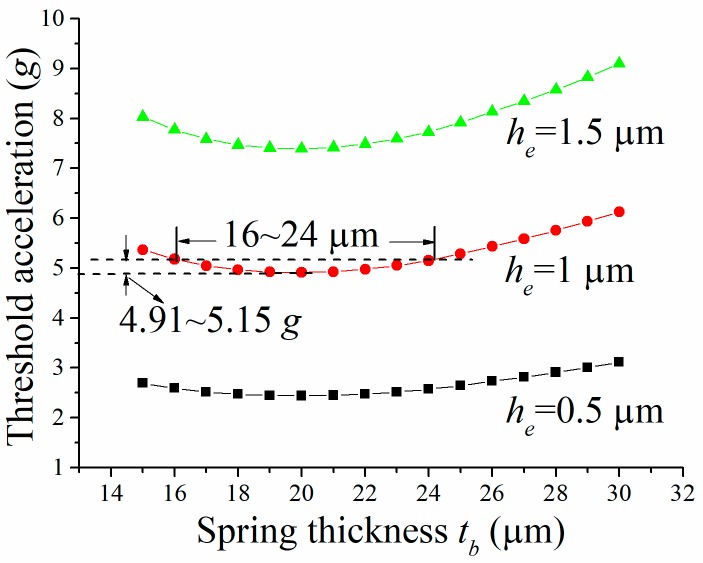
Threshold acceleration changes with the spring thickness in cases of different electrode-gap heights.

Figure 2 shows the changes of the threshold acceleration with the spring thickness $t_{b}$, for the cases when the electrode-gap height $h_{e}=$ 0.5 μm, 1 μm, and 1.5 μm, respectively. It can be seen that the threshold tends to level off when the value of $t_{b}$ changes around 20 μm, being in good agreement with the theoretical study. In the case of $h_{e}=$ 1 μm, the maximum threshold deviation is only 0.15 *g* (4.91–5.15 *g*), when $t_{b}$ increases from 16 to 24 μm. Since the 8 μm (16–24 μm) fabrication latitude is wide enough for the current bulk silicon micromachining technology, the threshold deviation of the switch caused by fabrication errors can be significantly reduced. As such, the values of $t_{b}$ ($t_{m}$) and $h_{e}$ were set to 20 μm and 1 μm, respectively.

## 3. Experiments and Discussion

**Figure 3:**
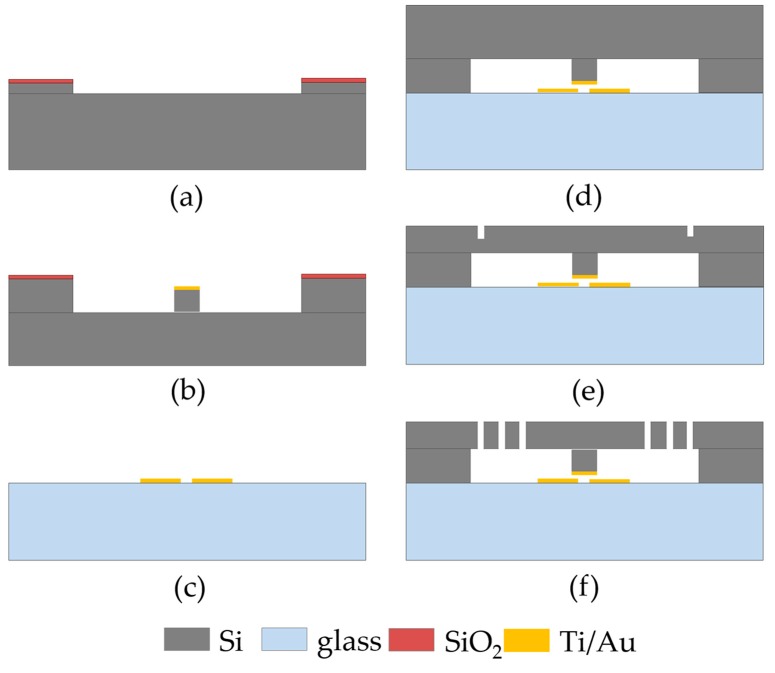
Process sequence for the fabrication of the micro-switch. (**a**) electrode gap; (**b**) protrusive electrode; (**c**) double-contact-configuration fixed electrode; (**d**) anodic bonding; (**e**) thinning and pre-release recess; (**f**) structure releasing.

**Figure 4:**
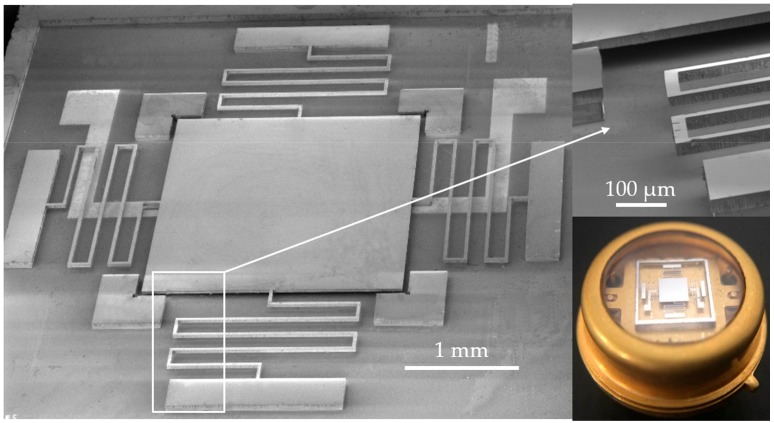
SEM (scanning electron microscope) and optical photographs of the fabricated and packaged micro-switches.

The switch was fabricated using a typical silicon-on-glass process. The silicon (<100> *n*-type) and glass wafers used in this work are both 4 inch in diameter and 500 μm in thickness. The main fabrication processes shown in Figure 3 have been presented in our previous study [22], except for a pre-release recess process (Figure 3e). Due to the anodic bonding process (Figure 3d), there is a pressure difference between the bonded chamber (10^−2^ mbar) and the outside air (ca. 1 bar), which may probably cause the protrusion to stick to the glass substrate, or even fracture the springs upon post-etch release of the structure (Figure 3f). Our experimental results show that this issue becomes evident as we reduce the electrode gap height from 2 μm to 1 μm. As such, several recesses (ca. 10 μm deep) on the edge of the chamber were constructed by an inductive plasma (ICP) etch (Figure 3e), which were firstly penetrated during the final structure release process (Figure 3f), thus eliminating the pressure difference. Figure 4 shows the fabricated and packaged micro-switches.

**Figure 5:**
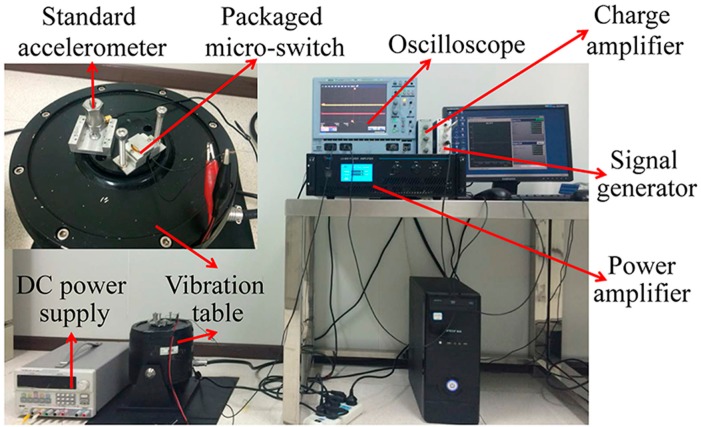
Experiment setup for testing the fabricated micro-switch.

**Figure 6:**
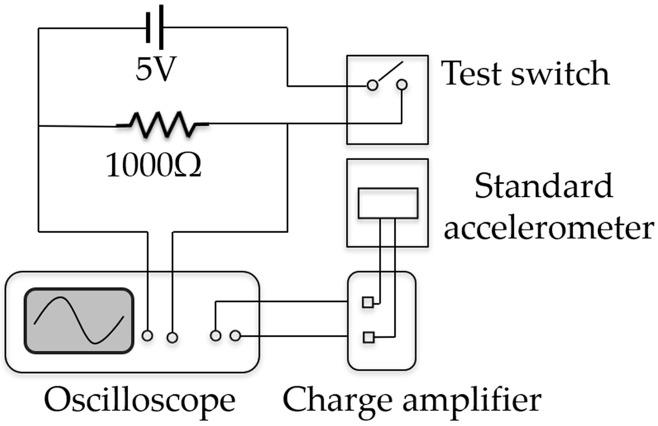
Schematic diagram of the test circuit.

The fabricated switch was tested by a vibration measurement system, as shown in Figure 5. Figure 6 shows the schematic of the test circuit. The packaged switch in a device holder was mounted on the vibration table, thus establishing a connection to a 5 V DC power supply, and a 1000 Ω resistor. A signal generator was used to offer a half-sine wave to simulate the acceleration load, which was amplified by a power amplifier and then fed into the vibration system to produce the required vibration. The vibration was detected by a standard accelerometer (100 mV/*g*) fixed on the vibration table beside the switch. The output of the accelerometer was amplified by a charge amplifier for facilitating the signal collection. The applied acceleration with various amplitudes (0.1~30 *g* with 0.1 *g* accuracy) and durations (>0.1 ms) was controlled by a computer. When the applied acceleration reaches the threshold value, the moveable electrode shorts the fixed electrode and thus turns on the test circuit, outputting voltage signal of ca. 5 V. The outputs of the switch and the accelerometer were recorded by a multichannel oscilloscope, and each experimental datum was repeated at least 10 times.

**Figure 7:**
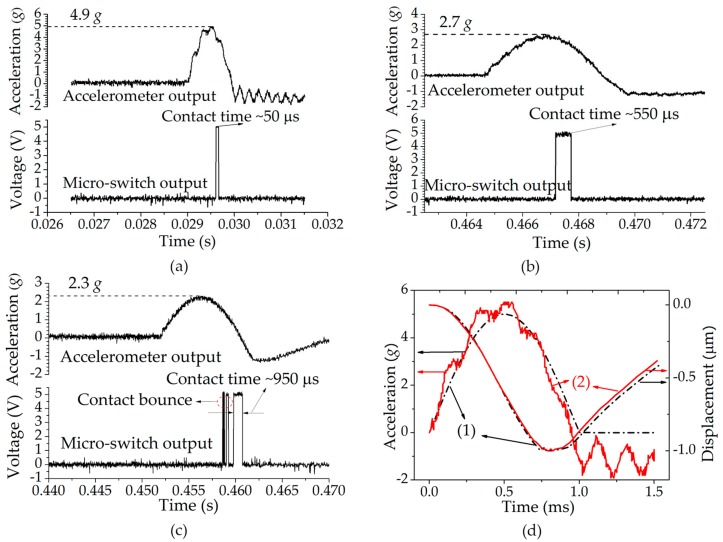
(**a**–**c**) The typical test results of the fabricated switches under (**a**) 1 ms, (**b**) 5 ms, and (**c**) 10 ms accelerations. (**d**) Simulation results of the displacement response of the switch under the cases of (1) a 30.9 μm spring width and a 5.5 *g* practical acceleration and (2) a 30 μm spring width and a 5 *g* standard half-sine wave.

Figure 7a shows the typical test results of the fabricated switches with a 4.9 *g* threshold under 1 ms acceleration duration. The threshold values of 10 randomly selected switches were tested as 4.9, 5.1, 5.2, 5.8, 5.3, 5.1, 5.5, 5.2, 5.5, and 5.6 *g* (hereafter, the switch is named after its threshold value under 1 ms acceleration), respectively. The SEM (scanning electron microscope, Carl Zeiss AG, Stuttgart, Baden-Württemberg, Germany) measurement results showed that the spring thicknesses between each switch range from 18.2 μm to 22.5 μm. The measured results were in accordance with the simulation results, and they are comparable to those of the switch based on a SOI wafer, wherein the device thickness can be accurately controlled [21]. Considering that the SOI wafer requires additional cost, compared with the common wafer used in this paper, the results show that the threshold accuracy enhancement presented in this paper is effective and low-cost.

The measured thresholds of the 4.9 *g* switch under 5 ms and 10 ms accelerations were 2.7 *g* and 2.3 *g*, respectively, as shown in Figure 7b,c. The test results of all 10 selected switches show that the thresholds were 2.2–3.8 *g* and 1.9–3.7 *g* for the cases of 5 ms and 10 ms accelerations, respectively, while their corresponding simulation results were 2.8 *g* and 2.6 *g*, respectively. It can be seen that with prolonging the acceleration duration, the measured threshold values were generally decreased, and the threshold deviation from the designed value became larger. Considering that the damping force that the switch is subjected to while operating is in proportion to the acceleration frequency as seen in Equation (8), the squeeze-film damping effect was obviously decreased, as the acceleration was prolonged from 1 ms to 5 ms, or 10 ms. Therefore, the lower threshold value and larger threshold deviation were both due to the weak squeeze-film damping effect, spotlighting the vital role of the squeeze-film damping on the improvement of threshold accuracy.

It should be noted that a higher threshold value measured under 1 ms acceleration was not necessarily relatively higher in the case of 5 ms or 10 ms acceleration. For example, under 10 ms acceleration, the measured threshold value of the 4.9 *g* switch was 2.7 *g*, which was lower than that of the 5.2 *g* switch (3.4 *g*), but was higher than that of the 5.1 *g* switch (2.3 *g*). This may be due to the randomness of the device thickness, leading to different squeeze-film damping effects, or it might be because unexpected fabrication errors.

Knowing that the variations of the measured spring thicknesses (18.2–22.5 μm) were all within the allowable latitude of 16–24 μm as obtained by the simulation, the threshold discrepancy in the measurement results between each device could contribute to the dimension errors of the spring width and the applied un-standard acceleration. In fact, the spring width of the fabricated switch was defined by the final structure release process by using an ICP etch. Since the silicon-on-glass bonded structure of the switch is similar to a SOI wafer, the etch profile of the springs is sensitive to the process parameters of the ICP etch, such as the RF power, the sample stage temperature, and the O_2_ gas flow rate [28]. SEM measurements showed that the spring widths of the fabricated switches were in the range of 29.6–30.9 μm. In order to further investigate the impacts of the dimension errors of the spring width and the applied non-standard acceleration on the threshold value, the response of the switch with the measured dimension of the spring width (30.9 μm) and under the actual acceleration (extracted from the oscilloscope) was simulated by the ANSYS Workbench. Figure 7d compares the obtained results (the solid line) with that of the switch with the designed spring width of 30 μm and under the standard half-sine wave (the dotted line). As seen in the figure, the practical acceleration was much rougher than the standard wave, and the maximum amplitude of the practical acceleration (5.5 *g*) which was regarded as the threshold value was higher than that of the standard wave (5 *g*). The results indicate that the dimensions errors of the spring width and the applied un-standard acceleration may led to the increment of the threshold value by 0.5 *g*. In addition, some other factors such as the residual stresses in the springs might also influence the threshold value.

The measured contact time of the 4.9 switch under 1 ms, 5 ms, and 10 ms accelerations was 50 μs, 550 μs and 950 μs, respectively, as seen in Figure 7a–c. The results were in good agreement with our previous study [22], and they were significantly longer than the typical switches with rigid electrodes, based on bulk silicon micromachining technology (usually less than 20 μs) [29,30]. The contact bounce occurring in Figure 7c was due to the rigid contact process of the two electrodes when the squeeze-film damping effect was weak [31].

## 4. Conclusions

In this work, the impact of squeeze-film damping on the threshold of a 5 *g* inertial micro-switch was studied by theoretical analysis, FEM simulation, and experimental test, based on our previous research. The theoretical analysis results indicate that the threshold variation due to the fabrication errors can be reduced by establishing a tradeoff between the damping and elastic forces. The design strategy was achieved by optimizing the device thickness (the thickness of the proof mass and springs) and verified by a FEM simulation. The simulation results show that the maximum threshold deviation was only 0.15 *g* when the variation range of the device thickness was 16–24 μm, which is an adequate wide latitude for the current bulk silicon micromachining technology. The switch was fabricated by a typical silicon-on-glass process and tested by a vibration measurement system. The test results indicate that the threshold values under 1 ms acceleration were between 4.9–5.8 *g*, and the device thicknesses were 18.2–22.5 μm. The enhanced threshold accuracy is comparable to that of the switch fabricated on a SOI wafer, wherein the device thickness can be accurately controlled. The threshold accuracy was generally decreased when the acceleration duration was prolonged from 1 ms to 5 ms, and then to 10 ms, wherein the squeeze-film damping effect is obviously decreased, spotlighting the vital role of the squeeze-film damping on the improvement of threshold accuracy. The contact time was also significantly prolonged (50 μs), compared with the typical silicon-based switches (usually less than 20 μs), showing a good agreement with our previous study. The study is beneficial in various inertial micro-switches where low-g-sensing, long contact time, and high threshold accuracy are required.

## Figures and Tables

**Table 1:** Comparisons of the main research results reported in the past.

Switch Category	Enhancement Method	Fabrication Method	Designed Threshold	Measured Threshold	Contact Effect
Contact effect enhanced switch	Latching [7]	Bulk silicon	50.59 *g*	28–43.7 *g*	Keep closed
	Bi-stable [9]	Nickel electroplating	35 *g*	32.38 *g*	Keep closed
	Micro-fluidic [11]	Bulk silicon	9 *g*	8.525 *g*	Keep closed
	Flexible electrodes [14]	Nickel electroplating	500 *g*	466 *g*	390 μs
	Flexible electrodes [15]	Nickel electroplating	240 *g*	288 *g*	150 μs
	Flexible electrodes [16]	Nickel electroplating	38 *g*	32–38 *g*	230 μs
Threshold accuracy enhanced switch	Redundancy design [18]	Nickel electroplating	10–150 *g*	80–90% of the target	- ^1^
	Dimension modification [4]	Nickel electroplating	90–230 *g*	10–40 *g*	- ^1^
	Dimension compensation [20]	Nickel electroplating	38 *g*	35–40 *g*	102 μs
	SOI wafer [21]	Bulk silicon	5.5 *g*	4.77–5.97 *g*	- ^1^

^1^ The data was not presented in the paper.

**Table 2:** Main geometrical parameters of the designed switch.

Component	Geometric Parameter	Value (μm)
Proof mass	Length $l_{m}$	2300
	Width $w_{m}$	2300
	Thickness $t_{m}$	=$t_{b}$
Protrusion	Length $l_{p}$	50
	Width $w_{p}$	50
	Height $h_{p}$	34
Serpentine spring	Span beam length $l_{s}$	1600
	Connector beam length $l_{c}$	150
	Width $w_{b}$	30
	Thickness $t_{b}$	Variable
Electrode gap	Height $h_{e}$	Variable

**Table 3:** Main material properties of the device structure.

Material	Density	Young’s Modulus	Poisson’s Ratio
Silicon	2330 kg/m^3^	169 GPa	0.28
Glass	2200 kg/m^3^	70 GPa	0.17
